# Case Report: Primary Pleural Angiosarcoma in a Patient With Klippel-Trenaunay Syndrome

**DOI:** 10.3389/fgene.2022.792466

**Published:** 2022-01-28

**Authors:** Jing Xu, Mengyao Liu, Hongtu Yuan, Zengjun Liu, Dongyuan Zhu

**Affiliations:** ^1^ Rare Tumors Department, Shandong Cancer Hospital and Institute, Shandong First Medical University and Shandong Academy of Medical Sciences, Jinan, China; ^2^ Department of Pathology, Shandong Cancer Hospital and Institute, Shandong First Medical University and Shandong Academy of Medical Sciences, Jinan, China; ^3^ Tumor Research and Therapy Center, Shandong Provincial Hospital, Cheeloo College of Medicine, Shandong University, Jinan, China

**Keywords:** klippel-trenaunay syndrome, angiosarcoma, PIK3CA, somatic mutations, pleura

## Abstract

Klippel-Trenaunay syndrome (KTS) was demonstrated as a mosaic activating PIK3CA mutations related overgrowth syndrome. We present the first case of primary pleural angiosarcoma in a 17-year-old woman with a history of KTS. The combined targeted DNA and RNA sequencing revealed an activating mutation in PIK3CA in the tumor tissue. Our case suggested an association and perhaps a causal link between the two different PIK3CA-related genetic diseases.

## Introduction

Klippel–Trenaunay syndrome (KTS), characterized by three major manifestations: port wine stain, venous varicosity, and asymmetrical hypertrophy of the limbs, was firstly described by Klippel and Trenaunay in 1900 (Klippel and Trenaunay, 1900). One hundred years later, a number of overgrowth syndromes with distinct, but partially overlapping clinical findings with KTS have been identified. With the identification of somatic mutations in the phosphatidylinositol-4,5-bisphospate 3-kinase, catalytic subunit alpha (PIK3CA) gene in these syndromes, an umbrella term of “PIK3CA Related Overgrowth Spectrum (PROS)” was proposed (Keppler-Noreuil et al., 2015; Vahidnezhad et al., 2016). Although cancers have been reported in individuals with PROS, whether PROS predisposes to cancer remains unclear. Herein, we present the first case of primary pleural angiosarcoma in a 17-year-old woman with a history of KTS. Moreover, based on the targeted DNA and RNA sequencing of a panel of 1,084 genes, we firstly demonstrated a somatic gain-of-function mutation in the PIK3CA gene (p.E542K) in the tumor tissues.

## Case Report

**FIGURE 1:**
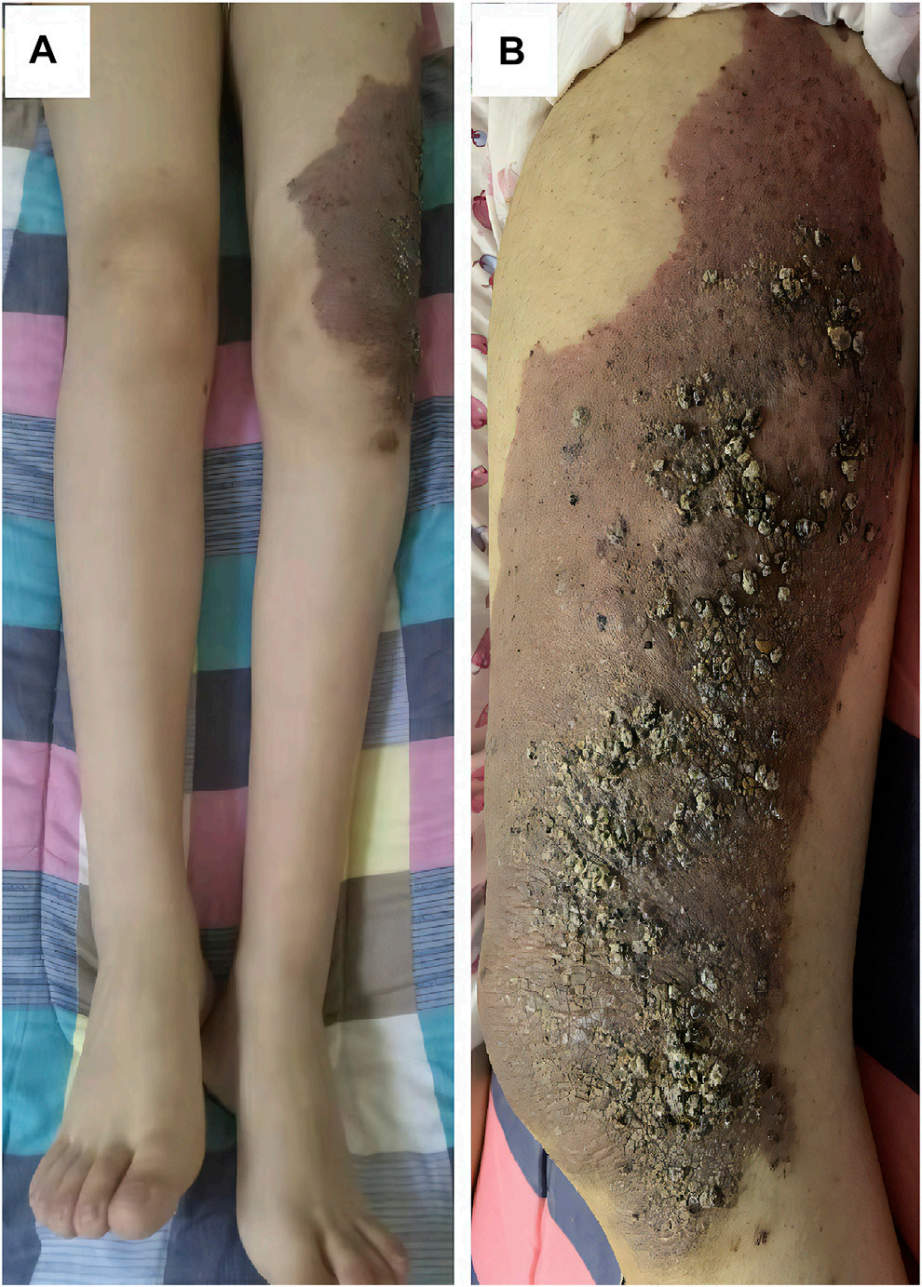
This patient had bone and soft-tissue hypertrophy **(A)**, port wine stains **(B)** and varicosity **(B)** involving her left thigh.

**FIGURE 2:**
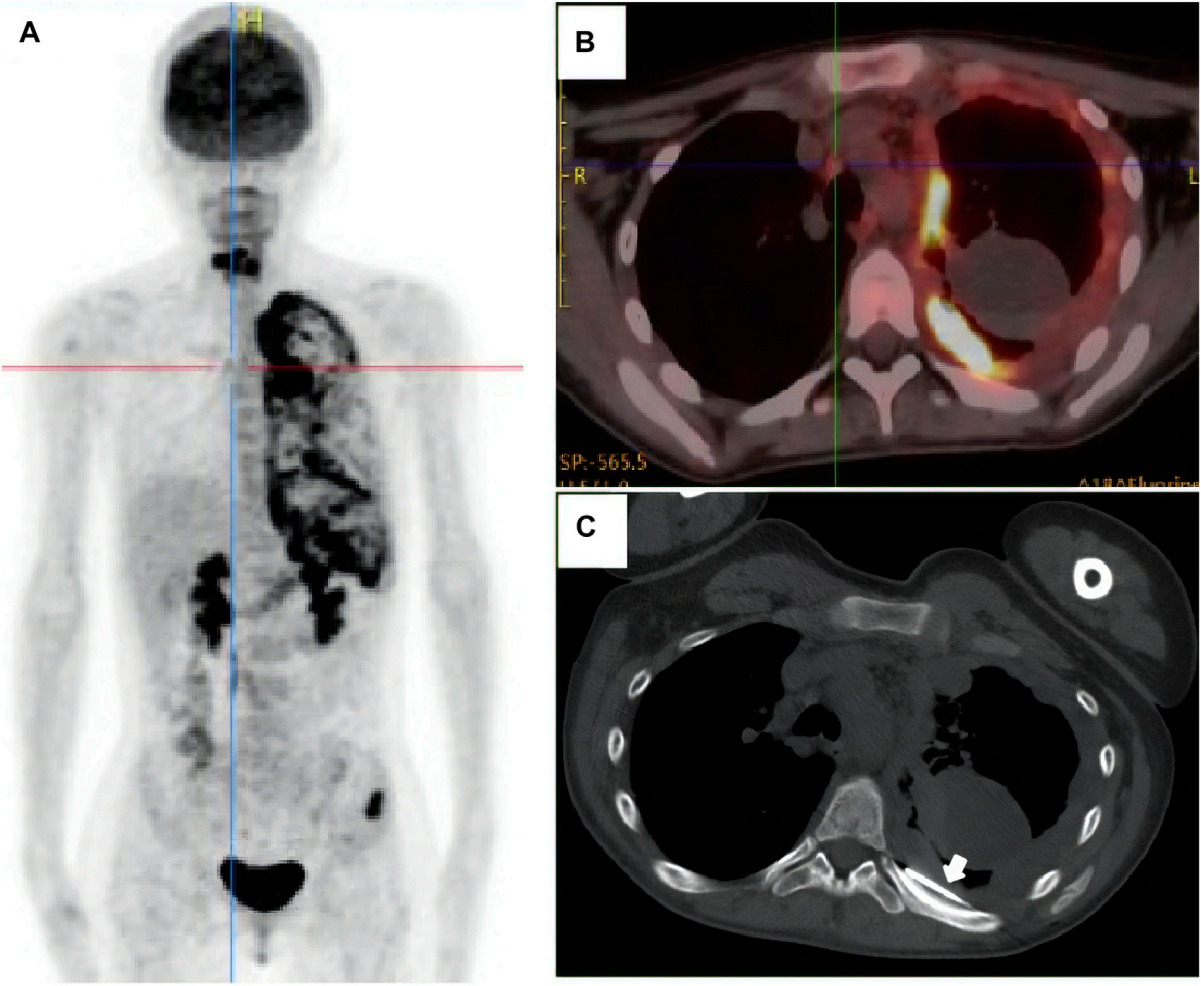
The positron emission tomography/computed tomography scan showed increased metabolic activity in the left iliac bone, the 6th cervical vertebra and the whole left pleura **(A, B)**. The patient underwent CT-guided biopsy of the left pleural mass [**(C)**, arrow].

**FIGURE 3:**
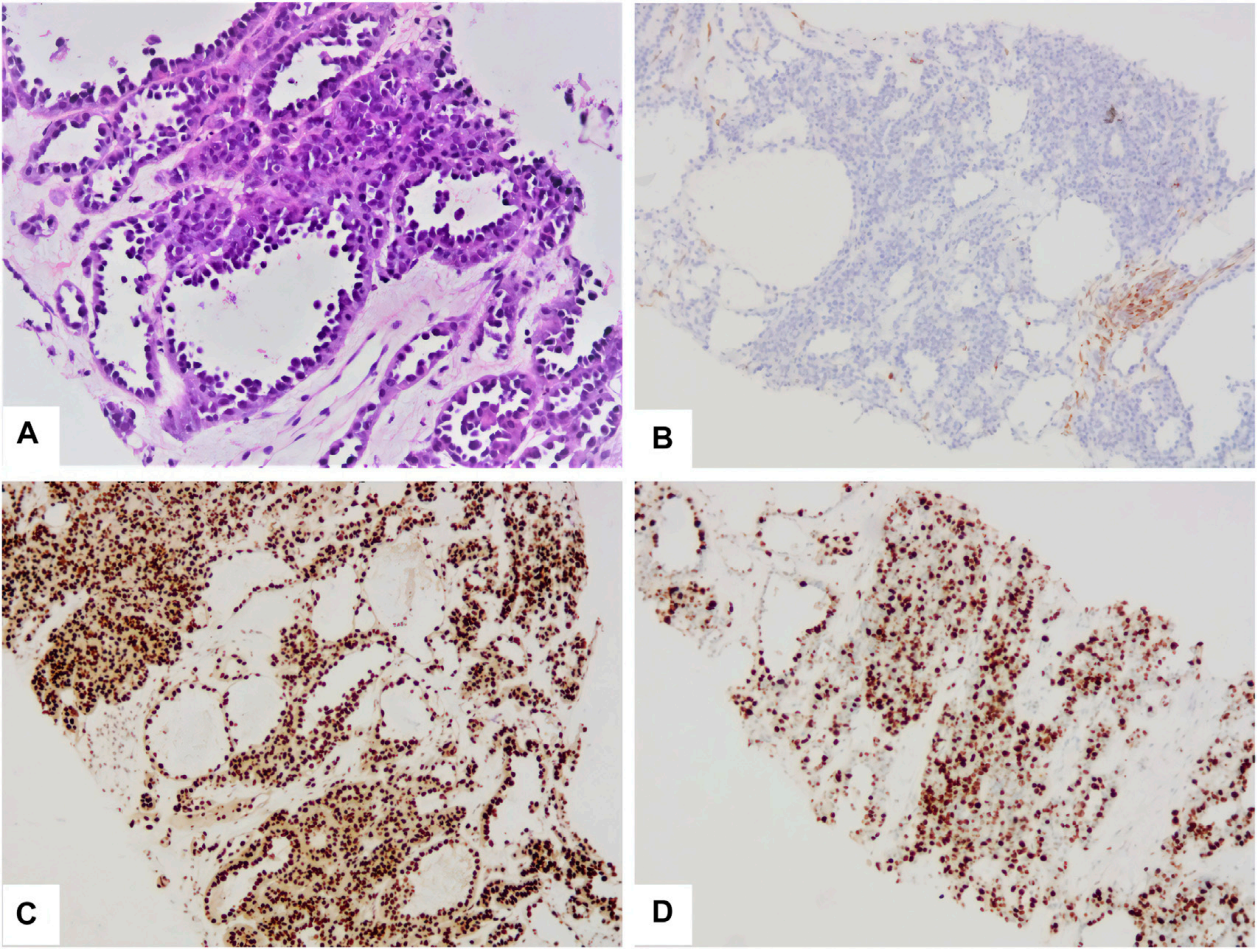
The tumor was composed of multiple irregular vascular lacunae **(A)**. The cavity was lined with hobnail tumor cells **(A)**. The tumor cells were negative for CKpan **(B)**, but positive for ERG **(C)**. The positive expression rate of ki67 was 60% **(D)**.

A 17-year-old woman presented to our rare tumors clinic complaining of a progressively increasing left thoracic pain for 4 months. She was diagnosed with KTS at the age of 5 based on a triad of port wine stains, varicosity, and bone and soft-tissue hypertrophy involving her left thigh (Figure 1). There is no evidence of vascular malformations or overgrowth anywhere else on the body. She reported no systemic disease. There was no history of similarly affected members within her family. A thoracic computed tomography (CT) scan revealed a moderate left pleural effusion. Malignant cells were identified in the exfoliative cytology of pleural effusion. The positron emission tomography/CT scan showed increased metabolic activity in the left iliac bone, the 6th cervical vertebra and the whole left pleura (Figure 2). CT-guided biopsy of the left pleural mass revealed a diagnosis of angiosarcoma. The tumor cells were positive for ERG, TLE1, P53, CD99 (focal), PLAP (weak), and CD117 (weak), but negative for CKpan, CK7, CK5, CK20, EMA, Oct4, P40, Villin, TTF-1, S-100, CEA, D2-40, CR, WT-1, Desmin, AFP, GPC3, and SALL4. The positive expression rate of ki67 was 60% (Figure 3). The laboratory tests revealed mild hypoalbuminemia and moderate anemia. The targeted DNA and RNA sequencing of a panel of 1,084 genes in paraffin-embedded tissue (for more detailed information, see Supplementary File S1 in the Supplementary Appendix) revealed only one single somatic mutation (PIK3CA p.E542K) in the tumor tissue. The mutant allele frequency is 34.3%. The tumor mutation burden is 0 mutations per megabase. The microsatellite stability analysis showed that the tumor was microsatellite stable.

**FIGURE 4:**
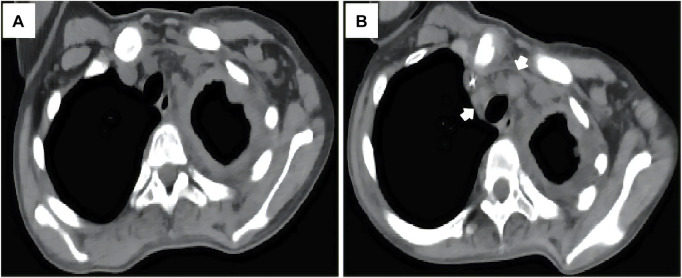
The patient experienced progressive disease during therapy, as indicated by the progression of the mediastinal lymph node metastases [**(A, B)**, arrow].

She accepted several cycles of therapy with nab-paclitaxel, pegylated liposomal doxorubicin, bevacizumab, eribulin, anlotinib (a multitargeted angiogenesis  inhibitor), everolimus, sintilimab (a humanized programmed death receptor-1 monoclonal antibody) and thalidomide successively within 4 months. However, unfortunately, the patient complained of progressively worsening left thoracic pain throughout the treatment. Progressive disease was confirmed during follow up (Figure 4). She died of dyspnea 6 months after the diagnosis.

## Discussion

The present case was diagnosed as KTS at the age of 5 based on the vascular anomaly and hypertrophy of the left thigh. The PET-CT scan showed no hemimegalencephaly or spinal deformity, which is present in CLOVES (Congenital Lipomatous asymmetric Overgrowth of the trunk with lymphatic, capillary, venous, and combined-type Vascular malformations, Epidermal naevi, Scoliosis/Skeletal and spinal anomalies). In light of the risk of hemorrhage, she refused biopsy of lesions in the left thigh. Hence, without confirmation of PIK3CA mutations, we diagnosed her as presumptive PROS according to the diagnostic criteria (Keppler-Noreuil et al., 2015).

Although KTS share the same PIK3CA hot-spot mutations with various cancers, the risk of cancers in patient with KTS does not appear to be higher than in the general population (Blatt et al., 2019). Actually, previous studies have demonstrated that PIK3CA is a poor oncogene on its own and usually require cooperating genetic lesions to induce cancer (Arafeh and Samuels, 2019).

In 2019, a whole exome sequencing study of 47 angiosarcoma tumors revealed that activating mutations in PIK3CA were observed nearly exclusively in primary breast angiosarcoma (Painter et al., 2020). Intriguingly, none of the 8 unique PIK3CA mutations (p.R88Q, p.N107T, p.P124L, p.G914R, p.T957P, p.M1043I, p.M1043V, and p.N1044K) were at the canonical PIK3CA hotspot residues E542. In the present case, the targeted DNA and RNA sequencing of a large panel of 1084 genes identified only one driver mutation, i.e., PIK3CA p.E542K in the primary pleural angiosarcoma. The TMB was 0. However, as mentioned earlier, the PIK3CA mutation is not suffificient to induce cancer on its own, which raises the possibility that, in the angiosarcoma tissues, undetected cooperating genetic lesions may exist. Due to the shortage of biopsied tissues, further genetic evaluation is limited. In addition, there is another possibility that a single PIK3CA gene mutation may be sufficient to induce tumorigenesis in some specific tissues.

The patient underwent several cycles of medical treatment with chemotherapy drugs, mTOR inhibitor, immune checkpoint inhibitor, and angiogenesis inhibitors. Unfortunately, the left thoracic pain progressively worsened throughout the treatment. Since PIK3CA inhibitors are not available in the mainland of China, we can’t evaluate the efficacy of these drugs in our patient.

Altogether, we present a PIK3CA-driven primary pleural angiosarcoma in a KTS patient. To our knowledge, this is the first case to suggest an association and perhaps a causal link between the two different PIK3CA-related genetic diseases. Further studies are needed to elucidate the spatiotemporal dynamics of phosphoinositide 3-kinase (PI3K) signaling in these different PI3K-related genetic diseases.

## Data Availability

The original contributions presented in the study are included in the article/Supplementary Material, further inquiries can be directed to the corresponding authors.
