# Supplementary material for: Case Report: Primary Pleural Angiosarcoma in a Patient With Klippel-Trenaunay Syndrome
Source: Front Genet. 2022 Jan 28;13:792466. doi: 10.3389/fgene.2022.792466 (PMC8831743; doi:10.3389/fgene.2022.792466)
Supplement: Supplementary file 1 [file Presentation1.PPTX]

## Slide 1
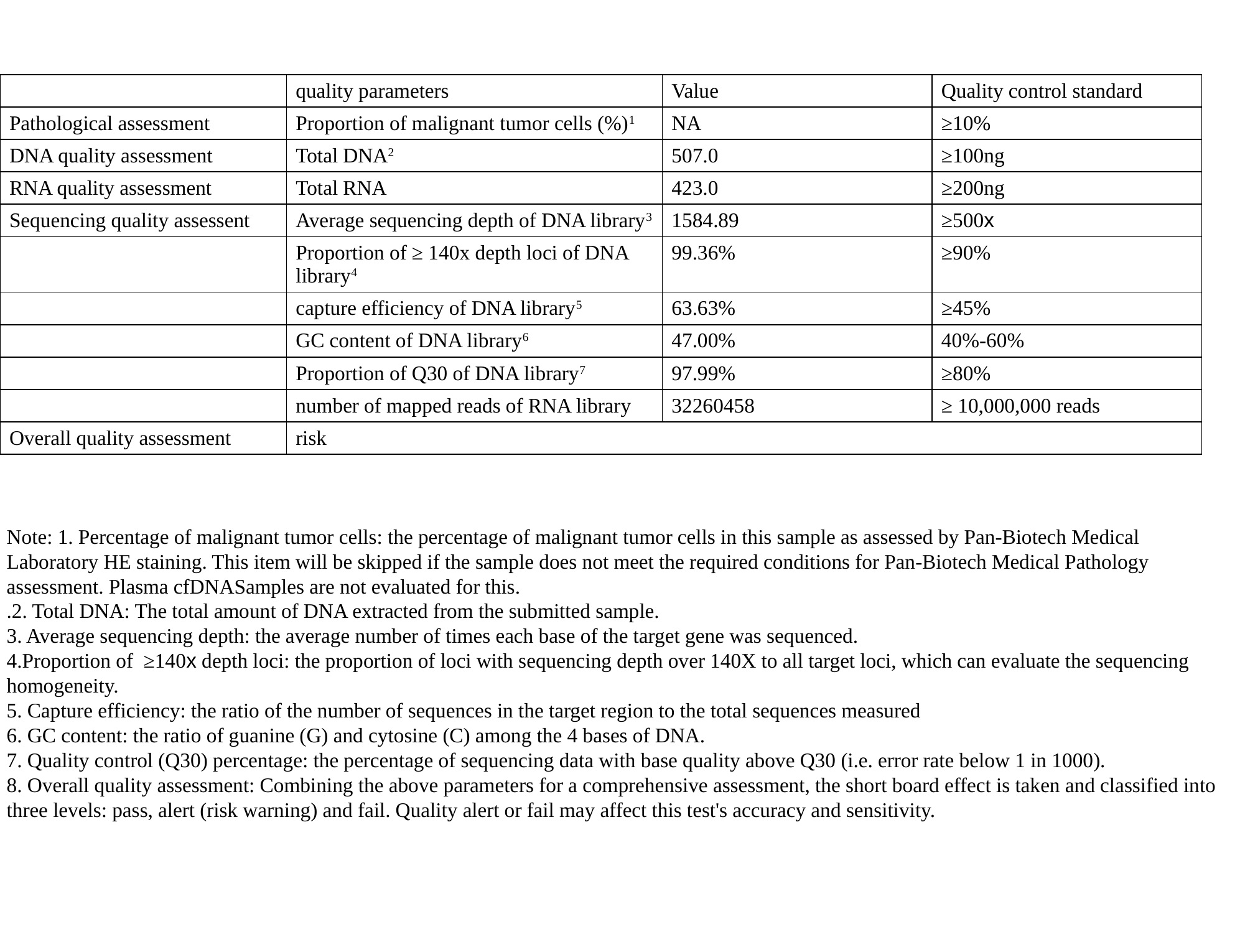

| | quality parameters | Value | Quality control standard |
| --- | --- | --- | --- |
| Pathological assessment | Proportion of malignant tumor cells (%)1 | NA | ≥10% |
| DNA quality assessment | Total DNA2 | 507.0 | ≥100ng |
| RNA quality assessment | Total RNA | 423.0 | ≥200ng |
| Sequencing quality assessent | Average sequencing depth of DNA library3 | 1584.89 | ≥500x |
| | Proportion of ≥ 140x depth loci of DNA library4 | 99.36% | ≥90% |
| | capture efficiency of DNA library5 | 63.63% | ≥45% |
| | GC content of DNA library6 | 47.00% | 40%-60% |
| | Proportion of Q30 of DNA library7 | 97.99% | ≥80% |
| | number of mapped reads of RNA library | 32260458 | ≥ 10,000,000 reads |
| Overall quality assessment | risk | | |
Note: 1. Percentage of malignant tumor cells: the percentage of malignant tumor cells in this sample as assessed by Pan-Biotech Medical Laboratory HE staining. This item will be skipped if the sample does not meet the required conditions for Pan-Biotech Medical Pathology assessment. Plasma cfDNASamples are not evaluated for this.
.2. Total DNA: The total amount of DNA extracted from the submitted sample.
3. Average sequencing depth: the average number of times each base of the target gene was sequenced.
4.Proportion of ≥140x depth loci: the proportion of loci with sequencing depth over 140X to all target loci, which can evaluate the sequencing homogeneity.
5. Capture efficiency: the ratio of the number of sequences in the target region to the total sequences measured
6. GC content: the ratio of guanine (G) and cytosine (C) among the 4 bases of DNA.
7. Quality control (Q30) percentage: the percentage of sequencing data with base quality above Q30 (i.e. error rate below 1 in 1000).
8. Overall quality assessment: Combining the above parameters for a comprehensive assessment, the short board effect is taken and classified into three levels: pass, alert (risk warning) and fail. Quality alert or fail may affect this test's accuracy and sensitivity.
