# Supplementary material for: Case Report: Primary Pleural Angiosarcoma in a Patient With Klippel-Trenaunay Syndrome
Source: Front Genet. 2022 Jan 28;13:792466. doi: 10.3389/fgene.2022.792466 (PMC8831743; doi:10.3389/fgene.2022.792466)
Supplement: Supplementary file 2 [file Table1.DOC]

**File 1: Target gene sequencing for sarcoma patients**

1. **The point mutations, insertions, and deletion analysis of 830 genes.**

A2M, ABCB4, ABL1, ABL2, ACTL6A, ACTL6B, ACVR1, ACVR1B, ACVR2A, AGO2, AIP, AJUBA, AKAP9, AKT1, AKT2, AKT3, ALB, ALK, ALOX12B, AMER1, ANGPT1, ANGPT2, ANKRD11, APC, APCDD1, APLNR, APOB, AR, ARAF, ARFRP1, ARID1A, ARID1B, ARID2, ARID5B, ASCL2, ASCL4, ASXL1, ASXL2, ATF1, ATIC, ATM, ATR, ATRX, AURKA, AURKB, AXIN1, AXIN2, AXL, B2M, B4GALT3, BABAM1, BACH1, BAI1, BAI2, BAI3, BAK1, BAP1, BARD1, BBC3, BCL10, BCL2, BCL2A1, BCL2L1, BCL2L11, BCL2L2, BCL6, BCOR, BCORL1, BCR, BIRC3, BLM, BMPR1A, BRAF, BRCA1, BRCA2, BRD4, BRD7, BRIP1, BTG1, BTG2, BTK, BUB1B, C1QA, C1R, C1S, CALR, CARD11, CARM1, CASP8, CBFB, CBL, CBLB, CBR1, CCND1, CCND2, CCND3, CCNE1, CD22, CD274, CD276, CD70, CD74, CD79A, CD79B, CDC25C, CDC42, CDC73, CDH1, CDH23, CDK12, CDK2, CDK4, CDK6, CDK8, CDKN1A, CDKN1B, CDKN1C, CDKN2A, CDKN2B, CDKN2C, CDX2, CEBPA ,CENPA, CFH, CFHR1, CFHR2, CFLAR, CHD2, CHD4, CHD7, CHEK1, CHEK2, CHUK, CIC, CRBN, CREBBP, CRIPAK, CRKL, CRLF2, CROT, CSDE1, CSF1R, CSF3R, CTCF, CTLA4, CTNNA1, CTNNB1, CUL3, CUL4A, CUL4B, CXCR4, CYLD, CYP17A1, CYSLTR2, DAPK1, DAXX, DCUN1D1, DDB2, DDR1, DDR2, DDX3X, DICER1, DIS3, DIS3L2, DNAJB1, DNMT1, DNMT3A, DNMT3B, DOT1L, DPYD, DROSHA, DUSP4, DUSP6, E2F3, EDNRA, EED, EGFL7, EGFR, EGR3, EIF1AX, EIF4A2, EIF4E, ELAC2, ELANE, ELF3, EML4, EMSY, EP300, EPAS1, EPCAM, EPHA2, EPHA3, EPHA4, EPHA5, EPHA7, EPHB1, EPHB2, EPHB4, EPHB6, ERBB2, ERBB3, ERBB4, ERCC1, ERCC2, ERCC3, ERCC4, ERCC5, ERF, ERG, ERRFI1, ESR1, ETV1, ETV4, ETV5, ETV6, EWSR1, EXT1, EXT2, EZH1, EZH2, EZR, F8, FAM135B, FAM175A, FAM46C, FAM58A, FANCA, FANCB, FANCC, FANCD2, FANCE, FANCF, FANCG, FANCI, FANCL, FANCM, FAS, FAT1, FAT3, FAT4, FBXW7, FCGR1A, FCGR2A, FCGR2B, FCGR2C, FCGR3A, FCGR3B, FGA, FGF10, FGF12, FGF14, FGF19, FGF23, FGF3, FGF4, FGF6, FGF7, FGFR1, FGFR2, FGFR3, FGFR4, FH, FHIT, FLCN, FLT1, FLT3, FLT4, FNTA, FOXA1, FOXA2, FOXL2, FOXO1, FOXP1, FPGS, FRK, FUBP1, FYN, FZR1, GAB2, GABRA6, GALNT12 GATA1 GATA2, GATA3, GATA4, GATA6, GDF1, GDF15, GEN1, GID4, GJB2, GLI1, GLI3, GNA11, GNA13, GNAQ, GNAS, GNRHR, GPC3, GPR101, GPR124, GPS2, GREM1, GRIN2A, GRM3, GSK3B, GSTT1, H3F3A, H3F3B, H3F3C, HCK, HDAC1, HDAC2, HDAC3, HDAC4, HDAC8, HDAC9, HES1, HGF, HIF1A, HIST1H1C, HIST1H2BD, HIST1H3A, HIST1H3B, HIST1H3C, HIST1H3D, HIST1H3E, HIST1H3F, HIST1H3G, HIST1H3H, HIST1H3I, HIST1H3J, HIST2H3C, HIST2H3D, HIST3H3, HLA-A, HLA-B, HLA-C, HMBS, HNF1A, HNF4A, HOXB13, HRAS, HRH2, HSD17B3, HSD3B1, HSD3B2, HSP90AA1, HSPA4, HUWE1, ICOSLG, ID3, IDH1, IDH2, IFNAR1, IFNAR2, IFNGR1, IFNGR2, IGF1, IGF1R, IGF2, IGF2R, IKBKB, IKBKE, IKZF1, IL10, IL6R, IL6ST, IL7R, INHA, INHBA, INPP4A, INPP4B, INPPL1, INSR, IRF2, IRF4, IRS1, IRS2, ITGB2, ITK, JAK1, JAK2, JAK3, JUN, KAT6A, KDM5A, KDM5C, KDM6A, KDR, KEAP1, KEL, KIF1B, KIF5B, KIT, KLF4, KLF5, KLHL6, KMT2A, KMT2B, KMT2C, KMT2D, KMT2E, KNSTRN, KRAS, LASP1, LATS1, LATS2, LCK, LIMK1, LMO1, LRP1B, LTK, LYN, LZTR1, MACF1, MAF, MALT1, MAML1, MAP2K1, MAP2K2, MAP2K3, MAP2K4, MAP3K1, MAP3K13, MAP3K14, MAPK1, MAPK3, MAPK8, MAPK8IP1, MAPKAP1, MAX, MC1R, MCL1, MDC1, MDM2, MDM4, MECOM, MED12, MEF2B, MEN1, MERTK, MET, MGA, MITF, MKNK1, MLH1, MLH3, MPL, MRE11A, MS4A1, MSH2, MSH3, MSH4, MSH5, MSH6, MSI1, MSI2, MSR1, MST1, MST1R, MTAP, MTOR, MTUS1, MUC1, MUTYH, MYB, MYBL1, MYC, MYCL, MYCL1, MYCN, MYD88, MYH9, MYOD1, NAB2, NAT1, NAT2, NBN, NCOA3, NCOR1, NCOR2, NEGR1, NEK11, NF1, NF2, NFATC2, NFE2L1, NFE2L2, NFE2L3, NFKBIA, NKX2-1, NKX3-1, NOTCH1, NOTCH2, NOTCH3, NOTCH4, NPM1, NR3C1, NRAS, NRG1, NRG3, NSD1, NT5C2, NTHL1, NTRK1, NTRK2, NTRK3, NUBPL, NUF2, NUP93, NUTM1, P2RY8, PAK1, PAK3, PAK7, PALB2, PALLD, PARK2, PARP1, PARP2, PARP3, PARP4, PAX5, PBRM1, PCBP1, PDCD1, PDCD1LG2, PDE11A, PDE4DIP, PDGFRA, PDGFRB, PDK1, PDPK1, PGR, PHF20L1, PHF6, PHOX2B, PIGF, PIK3C2B, PIK3C2G, PIK3C3, PIK3CA, PIK3CB, PIK3CD, PIK3CG, PIK3R1, PIK3R2, PIK3R3, PIM1, PLCG1, PLCG2, PLK1, PLK2, PMAIP1, PMS1, PMS2, PNRC1, POLD1, POLE, POLH, POT1, PPARG, PPM1D, PPP2R1A, PPP2R2A, PPP4R2, PPP6C, PRDM1, PRDM14, PREX2, PRKAA1, PRKACA, PRKACB, PRKAR1A, PRKCA, PRKCB, PRKCG, PRKCI, PRKD1, PRKDC, PROKR2, PRSS1, PRSS8, PSMB1, PSMB2, PSMB5, PTCH1, PTCH2, PTEN, PTK2, PTP4A1, PTP4A3, PTPN11, PTPN13, PTPRB, PTPRD, PTPRO, PTPRS, PTPRT, QKI, RAB35, RAC1, RAC2, RAD21, RAD50, RAD51, RAD51B, RAD51C, RAD51D, RAD52, RAD54L, RAF1, RARA, RARB, RARG, RASA1, RB1, RBL1, RBM10, RECQL, RECQL4, REL, RELA, RET, RFWD2, RGPD3, RHBDF2, RHEB, RHOA, RICTOR, RIT1, RNASEL, RNF43, ROBO1, ROCK1, ROS1, RPA1, RPL22, RPL5, RPS14, RPS6KA3, RPS6KA4, RPS6KB1, RPS6KB2, RPTOR, RRAGC, RRAS,

RRAS2, RSPO2, RTEL1, RUNX1, RUNX1T1, RUNX3, RXRA, RXRB, RXRG, RYBP, SBDS, SDC4, SDHA, SDHAF2, SDHB, SDHC, SDHD, SEMA3A, SEMA3E, SERPINB3, SERPINB4, SESN1, SESN2, SESN3, SETBP1, SETD2, SETD8, SF1, SF3B1, SGK1, SH2B3, SH2D1A, SHOC2, SHQ1, SIX1, SLAMF7, SLC34A2, SLC4A1, SLIT2, SLX4, SMAD2, SMAD3, SMAD4, SMAD7, SMARCA1, SMARCA2, SMARCA4, SMARCAL1, SMARCB1, SMARCC1, SMARCC2, SMARCD1, SMARCE1, SMC1A, SMC3, SMCHD1, SMO, SMYD3, SNCAIP, SOCS1, SOS1, SOX10, SOX17, SOX2, SOX9, SPEN, SPOP, SPRED1, SPRY4, SPTA1, SRC, SRCAP, SRD5A2, SRSF2, SSTR2, STAG2, STAT1, STAT2, STAT3, STAT4, STAT5A, STAT5B, STAT6, STK11, STK19, STK40, SUFU, SUZ12, SYK, TACC3, TAF1, TAP1, TAP2, TBL1XR1, TBX3, TCEB1, TCF12, TCF3, TCF7L2, TEK, TERC, TERT, TET1, TET2, TFG, TGFBR1, TGFBR2 ,THADA, TIPARP, TLR4, TMEM127, TMPRSS2, TNFAIP3, TNFRSF14, TNFRSF8, TNFSF11, TNFSF13B, TOP1, TOP2A, TOP3A, TP53, TP53BP1, TP63, TP73, TPM3, TPMT, TRAF2, TRAF7, TRPS1, TRRAP, TSC1, TSC2, TSHR,TSHZ2, TTF1, TUBA1A, TUBB, TUBD1, TUBE1, TUBG1, TYR, TYRO3, U2AF1, UGT1A1, UPF1, UROD, USHBP1, USP12, USP48, USP8, VEGFA, VEGFB, VEZF1, VHL, VTCN1, WAS, WEE1, WHSC1, WHSC1L1, WISP3, WNT10A, WNT10B, WNT7B, WRN, WT1, WWTR1, XIAP, XPA, XPC, XPO1, XRCC1, XRCC2, XRCC3, YAP1, YES1, ZFHX3, ZNF148, ZNF217, ZNF521, ZNF703, ZNRF3, ZRSR2.

1. **The rearrangement analysis of 44 genes.**

ALK, BCL2, BCR, BRAF, BRCA1, BRCA2, C19MC, CD74, EGFR, ETV4, ETV5, ETV6, EWSR1, EZR, FGFR1, FGFR2, FGFR3, KIT, KMT2A, MET, MSH2, MYB, MYBL1, MYC, NAB2, NOTCH2, NRG1, NTRK1, NTRK2, NTRK3, NUTM1, PDGFRA, PRKACA, RAF1, RARA, RELA, RET, ROS1, RSPO2, SDC4, SLC34A2, TERT, TMPRSS2, YAP1.

1. **The copy number variant analysis of 88 genes.**

ABL1, AKT1, AKT2, ALK, AR, ASCL2, BCL2, BRAF, C19MC, CBL, CCND1, CCNE1, CDK4, CDK6, CDK8, CSF1R, CTNNB1, DNMT3A, EGFR, ERBB2, ERBB3, ERBB4, EZH2, FGF19, FGF3, FGF4, FGFR1, FGFR2, FGFR3, FGFR4, FLT3, FOXL2, GATA2, GNA11, GNAQ, GNAS, H3F3A, HGF, HNF4A, HRAS, IDH1, IDH2, IGF1R, IGF2R, JAK1, JAK2, JAK3, KDR, KIT, KMT2A, KRAS, MAML1, MAP2K1, MDM2, MDM4, MED12, MET, MPL, MYB, MYC, MYCL, MYCN, MYD88, NCOA3, NKX2-1, NOTCH1, NOTCH2, NOTCH3, NOTCH4, NRAS, PAX5, PDGFRA, PIK3CA, PTPN11, RET, RICTOR, RUNX1, SDHA, SERPINB3, SERPINB4, SF3B1, SMO, TGFBR1, TOP3A, TSHR, USP12, VEGFA, WHSC1L1.

1. **The rearrangement analysis of 395 genes by RNA sequencing.**

ABI1, ABL1, ABL2, ACSL6, ADCY9, AFF1, AFF3, AFF4, AKT3, ALK, ARHGAP26, ARHGEF12, ARID1A, ARNT, ASXL1, ASXL2, ATF1, ATF7IP, ATG5, ATIC, AUTS2, AXL, BAALC, BCAT1, BCL10, BCL11A, BCL11B, BCL2, BCL3, BCL6, BCL7A, BCL9, BCOR, BCR, BIRC3, BLNK, BMF, BRAF, BTG1, BTK, C15ORF65, C1orf43, CAMTA1, CARS1, CBFA2T3, CBFB, CBL, CCNB3, CCND1, CCND2, CCND3, CD22, CD274, CD28, CD79B, CDK6, CDKN2A, CDX2, CEBPA, CEBPE, CEP85L, CHD1, CHIC2, CHMP2A, CHN1, CHST11, CIC, CIITA, CLP1, CLTC, CLTCL1, CNTRL, COL1A1, CREB1, CREB3L1, CREB3L2, CREBBP, CRLF2, CSF1, CSF1R, CSF3R, CTCF, CTLA4, CTNNB1, CUX1, DAZAP1, DDIT3, DDX10, DDX6, DEK, DENND3, DGKH, DNMT3A, DUSP22, DUX4, EBF1,

EGFR, EIF2B1, EIF4A2, ELF4, ELL, ELMO1, ELN, EMC7, EML4, ENTPD1, EP300, EPOR, EPS15, ERBB2, ERG, ETS1, ETV1, ETV4, ETV5, ETV6, EWSR1, FBN3, FBRSL1, FBXW2, FBXW7, FCGR2B, FCRL4, FER, FEV, FGFR1, FGFR1OP, FGFR2, FGFR3, FIP1L1, FLI1, FLT3, FLT3LG, FNBP1, FOXO1, FOXO3, FOXO4, FOXP1, FSTL3, FUS, FUT8, GAS7, GATA2, GLI1, GLIS2, GMPS, GPHN, GPI, HAVCR2, HERPUD1, HEY1, HIP1, H4C9, HLF, HMGA1, HMGA2, HNRNPUL1, HOXA10, HOXA11, HOXA13, HOXA3, HOXA9, HOXC11, HOXC13, HOXD11, HOXD13, HSP90AA1, HSP90AB1, IGHJ1, IKZF1, IKZF3, IL16, IL21R, IL2RB, IL3, IQGAP2, IRF4, IRS4, ITK, ITPKB, JAK1, JAK2, JAK3, JAZF1, KAT6A, KDM2B, KDM5A, KDM6A, KDSR, KIF5B, KLK11, KMT2A, KMT2D, KRAS, LASP1, LCK, LCP1, LMO1, LMO2, LPP, LRMP, LYL1, MAF, MAFB, MALT1, MAP2K4, MBNL1, MDS2, MECOM, MEF2D, MET, METRNL, MRTFA, MLF1, MLLT1, MLLT10, MLLT11, MLLT3, MLLT4, MLLT6, MME, MN1, MNX1, MSI2, MSN, MTCP1, MUC1, MYB, MYBL1, MYC, MYH11, MYH9, NAB2, NACA, NBEA, NCOA2, NDRG1, NEK6, NF1, NF2, NFKB2, NIN, NKX2-1, NONO, NOTCH1, NOTCH2, NPM1, NR4A3, NRAS, NSD1, NSD2, NTRK1, NTRK2, NTRK3, NUMA1, NUP214, NUP98, NUTM2A, OLIG2, OMD, P2RY8, PAFAH1B2, PAX3, PAX5, PAX7, PBX1, PCM1, PCSK7, PDCD1, PDCD1LG2, PDE4DIP, PDGFB, PDGFRA, PDGFRB, PER1, PHF1, PICALM, PIM1, PIM3, PLAG1, PML, POU2AF1, PPFIBP1, PPP1CB, PRDM1, PRDM16, PRRC1, PRRX1, PSIP1, PSMB2, PSMB4, PTCH1, PTK2B, PTK7, PTPN1, PUM1, PVT1, RAB7A, RABEP1, RAD51B, RAF1, RALGDS, RANBP2, RAP1GDS1, RARA, RB1, RBM15, RCSD1, REEP5, RET, RHOH, RNF213, ROS1, RPL22, RPN1, RUNX1, RUNX1T1, RUNX2, SEC31A, SEPT5, SEPT6, SEPT9, SERPINA9, SET, SETBP1, SF3B1, SH3BP5, SH3GL1, SKI, SLAMF7, SLC1A2, SNRPD3, SNX29, SPI1, SQSTM1, SRSF3, SS18, SS18L1, SSBP2, SSX1, SSX2, SSX4, STAG2, STAT5B, STAT6, STIL, STRN3, SYK, TAF15, TAL1, TAL2, TBL1XR1, TCF12, TCF3, TCF7, TCL1A, TEC, TET1, TFE3, TFG, TFPT, TFRC, TLX1, TLX3, TMPRSS2, TNFRSF11A, TNIP1, TOP1, TP53, TP63, TPM3, TPM4, TPR, TRIM24, TRIM27, TRIP11, TTL, TYK2, USP6, VCP, VPS29, WHSC1L1, WT1, WWTR1, XIAP, YPEL5, YWHAE, ZAP70, ZBTB16, ZCCHC7, ZEB2, ZFAND3, ZMYM2, ZNF384, ZNF521.

1. **The single nucleotide polymorphism analysis of 45 genes**

ABCB1 (rs1045642), ABCC4 (rs9561778), ATM (rs1801516), C8orf34 (rs1517114), CBR3 (rs1056892), CDA (rs2072671), CDA (rs60369023), CYP2B6 (rs3211371), DHFR (rs442767), DPYD (rs2297595), DPYD (rs3918290), DPYD (rs55886062), DPYD (rs67376798), DPYD (rs115232898), DPYD (rs17376848), DPYD (rs1801158), DPYD (rs1801159), DPYD (rs1801160), DPYD (rs1801265), DPYD (rs56038477), DYNC2H1 (rs716274), EGFR (rs2293347), EPHA5 (rs7349683), ERBB2 (rs1136201), ERBB3 (rs2229046), ERBB3 (rs773123), ERCC1 (rs11615), ERCC1 (rs3212986), ERCC2 (rs1052555), ERCC2 (rs13181), GGH (rs11545078), GSTP1 (rs1695), MTHFR (rs1801131), MTHFR (rs1801133), MTR (rs1805087), NAT2 (rs1799931), NAT2 (rs1801280), SLC22A16 (rs12210538), SOD2 (rs4880), TP53 (rs1042522), UGT1A1 (rs8175347), UMPS (rs1801019), VEGFA (rs25648), XPC (rs2228001),

XRCC1 (rs25487).

1. **A list of 148 susceptibility genes for cancers.**

AIP, AKT1, ALK, AMER1, APC, ATM, ATR, AXIN2, BAP1, BARD1, BLM, BMPR1A, BRCA1, BRCA2, BRIP1, BUB1B, CBL, CDC73, CDH1, CDK4, CDKN1B, CDKN1C, CDKN2A, CEBPA, CHEK1, CHEK2, CTNNB1, CYLD, DDB2, DICER1, DIS3L2, EGFR, ELANE, EPCAM, ERCC1, ERCC2, ERCC3, ERCC4, ERCC5, EXT1, EXT2, EZH2, FAM175A, FANCA, FANCB, FANCC, FANCD2, FANCE, FANCF, FANCG, FANCI, FANCL, FANCM, FAS, FH, FLCN, GALNT12, GATA2, GEN1, GPC3, GREM1, HMBS, HNF1A, HOXB13, HRAS, KIT, LASP1, MAX, MC1R, MEN1, MET, MITF, MLH1, MLH3, MRE11A, MSH2, MSH3, MSH6, MTAP, MTUS1, MUTYH, NBN, NF1, NF2, NSD1, NTHL1, NTRK1, PALB2, PALLD, PDE11A, PDGFRA, PHOX2B, PMS1, PMS2, POLD1, POLE, POLH, PPM1D, PRKAR1A, PRSS1, PTCH1, PTCH2, PTEN, PTPN11, RAD50, RAD51, RAD51B, RAD51C, RAD51D, RB1, RECQL, RECQL4, RET, RHBDF2, RUNX1, RUNX3, SBDS, SDHA, SDHAF2, SDHB, SDHC, SDHD, SLX4, SMAD4, SMARCA4, SMARCB1, SMARCE1, SOS1, STAT3, STK11, SUFU, TERT, TGFBR1, TGFBR2, TMEM127, TP53, TP63, TSC1, TSC2, UROD, USHBP1, VEGFA ,VHL, WRN, WT1, XPA, XPC, XRCC2.
